# Incidence and Predictors of Angiographic Vasospasm, Symptomatic Vasospasm and Cerebral Infarction in Chinese Patients with Aneurysmal Subarachnoid Hemorrhage

**DOI:** 10.1371/journal.pone.0168657

**Published:** 2016-12-15

**Authors:** Maimaitili Mijiti, Peierdun Mijiti, Aximujiang Axier, Maiwulanjiang Amuti, Zhu Guohua, Cheng Xiaojiang, Kaheerman Kadeer, Wang Xixian, Dangmurenjiafu Geng, Aisha Maimaitili

**Affiliations:** 1 Department of Neurosurgery, The First Affiliated Hospital of Xinjiang Medical University, Urumqi, Xinjiang, China; 2 Department of Epidemiology and Biostatistics, School of Public Health, Xinjiang Medical University, Urumqi, Xinjiang, China; 3 Department of Neurosurgery, The People's Hospital of Kashi, Kashi, Xinjiang, China; Universitatsklinikum Freiburg, GERMANY

## Abstract

**Introduction:**

Cerebral vasospasm (CVS) is the most common neurological complication after aneurysmal subarachnoid hemorrhage (aSAH) and associated with poor functional outcome and mortality. Reports on incidence and predictors of CVS in Chinese patients with aSAH were scarce. We aimed to estimate the incidence and predictors of angiographic vasospasm (AV), symptomatic vasospasm (SV), and cerebral infarction in Chinese patients with aSAH.

**Methods:**

We retrospectively reviewed the medical records of 542 consecutive aSAH patients admitted to neurosurgery department of the First Affiliated Hospital of Xinjiang Medical University in Urumqi city of China between January 1, 2011 and December 31, 2015. AV, SV and cerebral infarction were defined based on clinical data and neuroimaging findings. Univariate and multivariate analyses were performed to identify predictors of AV, SV or cerebral infarction.

**Results:**

343 (63.3%) patients fulfilled the inclusion and exclusion criteria. Of them, 182(53.1%) developed AV, 99 (28.9%) developed SV, and 87 (25.4%) developed cerebral infarction. A history of hypertension, poor modified Fisher grade (3–4) and poor Hunt-Hess grade (4–5) on admission were common risk factors for AV, SV and cerebral infarction. Patients from Uyghur ethnic group or other minorities were less likely to develop AV, SV or cerebral infarction, compared to those from Han ethic group after adjustment of other potential confounders. Additionally, age ≥53 years, leukocyte count ≥11× 10^9^/L on admission and being current or former smokers were independent risk factors of cerebral infarction. Leukocyte count ≥11× 10^9^/L on admission and aneurysm size ≥ 10 mm were independent risk factors of SV. Serum glucose level ≥7.0 mmol/L on admission was an independent risk factor of AV.

**Conclusion:**

Risk factors of different definitions of CVS were diverse in Chinese patients with aSAH; however, risk factors of SV and cerebral infarction seem to be similar. We recommend early and aggressive therapy in these patients at-risk of CVS.

## Introduction

Non-traumatic subarachnoid hemorrhage is a neurosurgical emergency characterized by the extravasation of blood into the spaces covering the central nervous system that are filled with cerebrospinal fluid. The leading cause of non-traumatic subarachnoid hemorrhage is rupture of an intracranial aneurysm, which accounts for about 80 percent of total cases [1]. Cerebral vasospasm (CVS) is the most common neurological complication after aneurysmal subarachnoid hemorrhage (aSAH) and associated with poor functional outcome and mortality [2, 3]. Understanding factors associated with development of CVS may help clinicians to target at-risk individuals and potentially prevent this complication. However, predictors of CVS after aSAH are reported inconsistently in previous literatures [4], which is mainly due to use of different definitions or diagnostic criteria for CVS. So far, terms used to define CVS have included angiographic vasospasm (AV), symptomatic vasospasm (SV), cerebral infarction on computed tomography (CT), delayed cerebral ischemia (DCI), and transcranial Doppler (TCD) vasospasm [5–9]. Recently, a multidisciplinary research group proposed a uniform definition of CVS, which recommended using separately defined outcomes for AV, clinical deterioration due to DCI, and cerebral infarction, rather than combining radiographic evidence of vasospasm with clinical features of cerebral ischemia [10–12]. TCD is used to monitor patients with aSAH for increases in intracranial blood flow velocity suggestive of incipient CVS. However, diagnostic performance of TCD at confirming CVS is still controversial [13–15]. Incidence and predictors of CVS after aSAH in patients with different ethnic background are well documented in the literatures [16–18]. However, reports on incidence and predictors of CVS in Chinese patients with aSAH are rare. In this study, we aimed to investigate the incidence and predictors of CVS defined by AV, or SV, or cerebral infarction in aSAH patients admitted to the First Affiliated Hospital of Xinjiang Medical University in Urumqi city of western China in 2011–2015.

## Materials and Methods

### Study patients

We retrospectively reviewed the demographic and clinical data from patients admitted to the Department of Neurosurgery at Xinjiang Medical University First Affiliated Hospital (Urumqi City, Xinjiang Province, China) with confirmed aSAH between January 1, 2011 and December 31, 2015. aSAH was determined by clinical symptoms and neuroimaging findings (digital subtract angiography [DSA], CT or CT angiography [CTA]). Lumbar puncture was performed when angiography could not confirm subarachnoid hemorrhage. Exclusion criteria included secondary subarachnoid hemorrhage from trauma, arteriovenous malformations, or other causes and age less than 15 years. Patients were only included when they met the following criteria: they were admitted to neurosurgery center within two days after aSAH onset, and received surgical clipping and/or endovascular coiling within three days after admission, and had repeated DSA performed after aneurysm treatment, and had CT scan performed on admission and repeated several times within six weeks after aSAH.

### Management of aSAH patients

Patients with aSAH were treated following a standardized institutional protocol. Upon admission, all patients were treated with intravenous fluids to maintain euvolemia. Oral nimodipine was administered to all patients (60 mg every four hours) for 14 days from the time of admission. For those who were not able to take oral nimodipine, intravenous nimodipine was administered. Selection of treatment with surgical clipping or endovascular coiling resulted from a consensus reached between the treating neurosurgeon and the interventional neuro-radiologist after analyzing risks and chances of success of both therapeutic modalities on each case. All patients underwent DSA or CTA, and CT scan on admission. Based on clinical symptoms and neuroimaging findings on admission, the clinical condition of aSAH patients was graded according to Hunt and Hess [19] and the severity of aSAH was radiologically classified according to Frontera JA, et al (modified Fisher grade) [20]. Patients usually had one DSA between three and five days after aneurysm treatment. Repeated DSA would be performed when the diagnosis of vasospasm remained in question or endovascular treatment was being entertained. Head CT scan was performed on as-needed basis for clinical purposes during the time between admission and discharge.

### Definitions of outcome variables

SV was defined as the development of new focal neurological signs within 21 days after aSAH onset, including worsening headaches, stiff neck and insidious onset of confusion, or decline in level of consciousness, or focal deficits not clinically or radiographically attributable to other causes (hydrocephalus, seizures, metabolic derangement, infection, etc.). AV was defined as moderate-to-sever arterial narrowing on DSA and not attributable to atherosclerosis, catheter-induced spasm or vessel hypoplasia. The degree of narrowing was determined by comparing the diameter of an affected artery to its pre-narrowed segment or to the most distal segment of the parental vessel. Narrowing was classified as moderate when the decrease was between 25% and 50% and severe when the vessel was narrowed by more than 50% [21]. Cerebral infarction was defined radiologically as a new hypodensity located in a vascular distribution on CT scan either within six weeks after SAH or before discharge and not attributable to other causes such as surgical clipping or endovascular treatment. In this study, hypodensities on CT scan resulting from ventricular catheter or intraparenchymal hematoma were not regarded as cerebral infarctions from DCI [11]. AV, SV, and cerebral infarction due to vasospasm were independently assessed without knowledge of each.

### Other variables used in the analysis

Other variables potentially associated with AV, SV (both transient and permanent), and cerebral infarctions were collected. Those variables included basic demographic information (age, gender, ethnicity, and history of cigarette smoking and hypertension), size and number of aneurysm, Hunt-Hess grades and modified fisher grades on admission, aneurysm treatment (surgical clipping and endovascular coiling) and admission laboratory findings (leukocyte count, serum glucose, and triglyceride). The age variable was dichotomized into “≥ 53 years” and “< 53 years” (the mean age was selected as the cutoff value). Aneurysm size was determined by measuring the maximal inner diameter on DSA and categorized into two groups: “size ≥ 10 mm” (at least one aneurysm size ≥ 10 mm for multiple aneurysms) and “size < 10 mm” (size of all aneurysms < 10 mm for multiple aneurysms). The Hunt-Hess and Modified Fisher grades on admission were dichotomized into good-grade status (Modified Fisher grade “0–2” or Hunt-Hess grade “1–3”) and poor-grade status (Modified Fisher grade “3–4” or Hunt-Hess grade “4–5”). Admission leukocyte count, serum glucose, and triglyceride were dichotomized clinically at the level of 11× 10^9^/L, 7.0 mmol/L and 2.26 mmol/L, respectively.

### Ethics statement

The Xinjiang Medical University First Affiliated Hospital Ethics Committee reviewed and approved the analysis. All private information was kept confidential and used for population analysis only.

### Statistical analysis

All statistical analyses were performed in SPSS version 17 (IBM Statistics, Chicago, IL). Univariate analysis was performed separately using AV, or SV, or cerebral infarction as the dependent variable. The demographic, clinical, radiographic, treatment-related, and in-hospital complication variables described above were independent variables. Categorical variables were analyzed using χ^2^ tests. The relative difference between outcomes was expressed as an odds ratios (ORs) with 95% confidence interval (CI). Binary logistic regression with stepwise forward selection was then used to adjust for variables identified in univariate analysis to be significantly different between the outcomes. The probability value for choice of predictive variables in logistic regression was set at 0.05. Adjusted ORs (aORs) with 95% CI were given. Independence of variables was tested using the likelihood ratio test on reduced models. Significance was set at *P* < 0.05 for all analyses.

## Results

**Fig 1:**
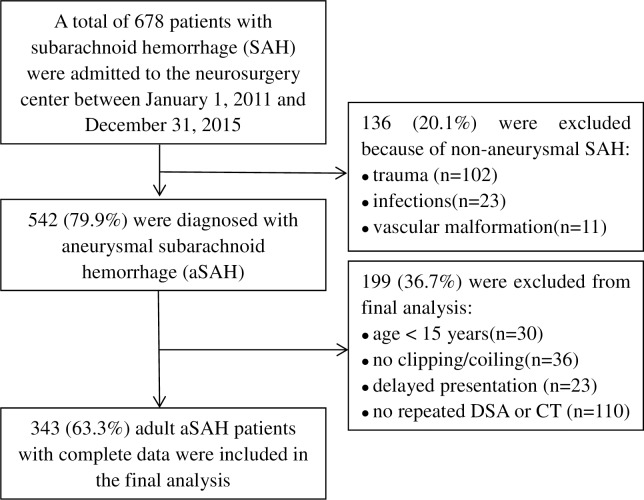
Flow diagram of the eligible patients and enrollment process of the study.

A total of 678 patients with subarachnoid hemorrhage were admitted to the Department of Neurosurgery at Xinjiang Medical University First Affiliated Hospital between January 1, 2011 and December 31, 2015. Of them, 542 patients were diagnosed with aSAH. A total of 343 aSAH patients (63.3%) met the inclusion and exclusion criteria, and were included in the final analysis (Fig 1). Demographic and clinical characteristics of these eligible aSAH patients are shown in Table 1. The median time interval of performing DSA after aneurysm treatment was five days (range from two days to seven days). Among 343 patients with aSAH, 182(53.1%) developed AV, 99 (28.9%) developed SV, and 87 (25.4%) developed cerebral infarction. Of those with AV, 88(48.4%) had SV and 70 (38.5%) had cerebral infarction. Of those with SV, 88(88.9%) had AV and 42 (42.4%) had cerebral infarction. 10 out of 87 patients with cerebral infarction (11.5%) showed no evidence of SV or AV.

**Table 1:** Demographic and clinical characteristics of 343 patients with aSAH on admission.

Category	Number	Proportion (%)
**Gender**		
**male**	133	38.8
**female**	210	61.2
**Age group**		
**≥53 years**	151	44.0
**< 53 years**	192	56.0
**Ethnicity**		
**Han**	191	55.7
**Uyghur**	92	26.8
**Others**	60	17.5
**History of cigarette smoking**		
**Current or former smokers**	64	18.7
**Non-smokers**	279	81.3
**History of hypertension**		
**Yes**	149	43.7
**No**	194	56.3
**Number of aneurysms**		
**1**	289	84.3
**≥2**	54	15.7
**Aneurysm size**		
**≥ 10 mm**	166	48.4
**< 10 mm**	177	51.6
**Modified Fisher grade**		
**0–2**	194	56.6
**3–4**	149	43.4
**Hunt-Hess grade**		
**1–3**	226	65.9
**4–5**	117	34.1
**Leukocyte count**		
**≥11×10**^**9**^**/L**	168	40.0
**< 11×10**^**9**^**/L**	137	49.0
**Missing data**	38	11.0
**Serum glucose level**		
**≥7.0 mmol/L**	156	45.5
**<7.0 mmol/L**	151	44.0
**Missing data**	36	10.5
**Triglyceride level**		
**≥2.26 mmol/L**	31	9.0
**< 2.26 mmol/L**	257	74.9
**Missing data**	55	16.1
**Aneurysm treatment**		
**Clipping**	216	63.0
**Coiling**	107	31.2
**Both**	20	5.8

In univariate analysis, age ≥ 53 years, poor modified Fisher grade (3–4), poor Hunt-Hess grade (4–5), leukocyte count level ≥11× 10^9^/L, and serum glucose level ≥7.0 mmol/L on admission were risk factors of AV, or SV, or cerebral infarction. Additionally, a history of hypertension and being current or former smokers were risk factors for SV or cerebral infarction. Aneurysm size ≥ 10 mm was risk factor for AV or SV (Table 2). Notably, aSAH patients from Uyghur ethnic group or other minority groups (Kazakh, Hui and Mongol) were less likely to develop AV, or SV, or cerebral infarction compared to those from Han ethnic group in univariate analysis.

**Table 2:** Univariate analysis of risk factors associated with occurrence of AV, SV and cerebral infarction in patients with aSAH.

Category	AV(n = 182)			SV(n = 99)			cerebral infarction (n = 87)		
	N (%)	OR (95% CI)	*P*	N (%)	OR (95% CI)	*P*	N (%)	OR (95% CI)	*P*
**Gender**									
**Female**	83(62.4)	1.9(1.2–2.9)	0.006	45(33.8)	1.5(0.9–2.4)	0.105	35(26.3)	1.1(0.7–1.8)	0.747
**Male**	99(47.1)	1.0		54(25.7)	1.0		52(24.8)	1.0	
**Age group**									
**≥53 years**	94 (62.3)	2.0(1.3–3.0)	0.002	59(39.1)	2.4(1.5–3.9)	<0.001	50(33.1)	2.1(1.3–3.4)	0.003
**< 53 years**	88 (45.8)	1.0		40(20.8)	1.0		37(19.3)	1.0	
**Ethnicity**									
**Han**	115 (60.2)	1.0		55(35.1)	1.0		66(34.6)	1.0	
**Uyghur**	45 (48.9)	0.6(0.4–1.0)	0.073	26(21.7)	0.5(0.3–0.9)	0.023	15(16.3)	0.4(0.2–0.7)	0.001
**Others**	22 (36.7)	0.4(0.2–0.7)	0.002	18(20.0)	0.5(0.2–0.9)	0.028	6(10.0)	0.2(0.1–0.5)	<0.001
**History of cigarette smoking**									
**Current or former smokers**	34 (53.1)	1.0(0.6–1.7)	0.991	31(48.4)	2.9(1.7–5.0)	<0.001	23(35.9)	1.9(1.1–3.4)	0.031
**Non-smokers**	148(53.1)	1.0		68(24.4)	1.0		64(22.9)	1.0	
**History of hypertension**									
**Yes**	79 (53.0)	1.0(0.6–1.5)	0.921	65(43.6)	3.8(2.4–6.2)	<0.001	47(31.5)	1.8(1.1–2.9)	0.021
**No**	103 (53.1)	1.0		34(16.8)	1.0		40(20.6)	1.0	
**Number of aneurysms**									
**1**	155(53.6)	1.0		84(27.4)	1.0		72(24.9)	1.0	
**≥2**	27(50.0)	0.9(0.5–1.5)	0.875	15(27.8)	1.0(1.0–2.3)	0.947	15(27.8)	1.2(0.6–2.2)	0.657
**Aneurysm size**									
**≥ 10 mm**	99(53.6)	1.7(1.1–2.6)	0.018	58(34.9)	1.8(1.1–2.9)	0.016	46(27.7)	1.3(0.8–2.1)	0.333
**< 10 mm**	83(46.9)	1.0		31(23.3)	1.0		41(23.2)	1.0	
**Modified Fisher grade**									
**0–2**	70 (36.1)	1.0		43(22.2)	1.0		34(17.5)	1.0	
**3–4**	112 (75.2)	5.4(3.4–8.5)	<0.001	56(37.6)	2.1(1.3–3.4)	0.002	53(35.6)	2.6(1.6–4.2)	<0.001
**Hunt-Hess grade**									
**1–3**	79 (35.0)	1.0		35(15.5)	1.0		30(13.3)	1.0	
**4–5**	103(88.0)	13.7(7.4–25.5)	<0.001	64(54.7)	6.6(4.1–10.7)	<0.001	57(48.7)	6.2(3.8–10.2)	<0.001
**Leukocyte count**									
**≥11×10**^**9**^**/L**	117(69.6)	2.5(1.6–4.0)	<0.001	65(38.7)	1.9(1.2–3.1)	0.010	61(36.3)	2.4(1.4–4.1)	0.001
**<11×10**^**9**^**/L**	65(47.4)	1.0		34(24.8)	1.0		26(19.0)	1.0	
**Serum glucose level**									
**≥7.0 mmol/L**	104(66.7)	3.2(2.0–5.1)	<0.001	63(40.4)	2.2(1.3–3.5)	0.002	49(31.4)	1.8(1.1–3.0)	0.030
**<7.0 mmol/L**	58(38.4)	1.0		36(23.8)	1.0		31(20.5)	1.0	
**Triglyceride level**									
**≥2.26 mmol/L**	16(51.6)	0.9(0.5–2.0)	0.891	10(32.3)	0.9(0.4–2.0)	0.793	11(35.5)	1.4(0.6–3.1)	0.386
**<2.26 mmol/L**	136(52.9)	1.0		89(34.6)	1.0		72(28.0)	1.0	
**Aneurysm treatment**									
**Clipping**	107(49.5)	1.0		68(31.5)	1.0		56(25.9)	1.0	
**Coiling**	66(61.7)	1.6(1.0–2.6)	0.042	25(23.4)	0.7(0.4–1.1)	0.129	25(23.4)	0.9(0.5–1.5)	0.951
**Both**	9(45.0)	0.8(0.3–2.1)	0.698	6(30.0)	0.9(0.3–2.5)	0.891	6(30.0)	1.2(0.4–3.3)	0.692

aSAH, aneurysmal subarachnoid hemorrhage; AV, angiographic vasospasm; SV, symptomatic vasospasm; OR, odds ratio; CI, confidence interval.

In multivariate analysis, ethnicity, history of hypertension, modified Fisher grade, and Hunt-Hess grade were associated with development of AV, or SV, or cerebral infarction. In addition, age, leukocyte count and history of cigarette smoking were associated with development of cerebral infarction. Leukocyte count and aneurysm size were associated with development of SV. Serum glucose level was associated with development of AV (Table 3). Gender, number of aneurysms, triglyceride level and aneurysm treatment were not associated with development of AV, or SV, or cerebral infarction.

**Table 3:** Multivariate analysis of risk factors associated with AV, SV and cerebral infarction in patients with aSAH.

Category	AV (n = 182)		SV (n = 99)		Cerebral infarction (n = 87)	
	aOR (95% CI)	*P*	aOR (95% CI)	*P*	aOR (95% CI)	*P*
**Age group**						
**≥53 years**	0.9(0.3–3.1)	0.922	0.8(0.5–1.1)	0.203	1.6(1.1–2.4)	0.042
**< 53 years**	1.0		1.0		1.0	
**Ethnicity**						
**Han**	1.0		1.0		1.0	
**Uyghur**	0.5(0.3–0.7)	0.003	0.6(0.3–0.9)	0.023	0.7(0.5–0.9)	0.043
**Others**	0.6(0.2–0.8)	0.005	0.4(0.2–0.6)	0.015	0.6(0.4–0.8)	0.032
**History of cigarette smoking**						
**Current or former smokers**	0.9(0.2–5.0)	0.987	2.4(0.6–4.2)	0.654	2.1(1.4–3.2)	0.021
**Non-smokers**	1.0		1.0		1.0	
**History of hypertension**						
**Yes**	2.1(1.3–3.9)	0.023	4.4(1.8–10.7)	<0.001	2.7(1.7–4.2)	<0.001
**No**	1.0		1.0		1.0	
**Aneurysm size**						
**≥ 10 mm**	2.2(0.5–9.3)	0.278	1.6(1.1–2.3)	0.021	1.0(0.5–1.9)	0.990
**< 10 mm**	1.0		1.0		1.0	
**Modified Fisher grade**						
**0–2**	1.0		1.0		1.0	
**3–4**	4.1(1.3–9.4)	<0.001	3.3(1.2–6.7)	<0.001	2.3(1.5–3.5)	<0.001
**Hunt-Hess grade**						
**1–3**	1.0		1.0		1.0	
**4–5**	7.9(2.5–13.3)	<0.001	6.5(2.2–10.8)	<0.001	5.3(1.3–7.9)	<0.001
**Leukocyte count**						
**≥11× 10**^**9**^**/L**	2.7(0.8–9.6)	0.126	2.2(1.2–5.7)	0.034	1.5(1.2–3.4)	0.007
**< 11× 10**^**9**^**/L**	1.0		1.0		1.0	
**Serum glucose level**						
**≥7.0 mmol/L**	3.9(1.1–13.9)	0.036	1.7(0.9–3.1)	0.107	1.3(0.8–2.2)	0.279
**<7.0 mmol/L**	1.0		1.0		1.0	

aSAH, aneurysmal subarachnoid hemorrhage; AV, angiographic vasospasm; SV, symptomatic vasospasm; CI, confidence interval; aOR, adjusted odds ratio.

## Discussion

To our knowledge, this is the largest study that investigates the incidence and predictors of AV, SV and cerebral infarction among Chinese patients with aSAH. The estimated incidence of AV, SV and cerebral infarction among patients with aSAH in our study is 53.1%, 28.9% and 25.4%, respectively. These findings are in alignment with the observed incidence between lowest and highest incidences reported by other studies [7, 16, 17, 22–26]. However, our results should be applied with caution due to the exclusion of nearly 37% of patients with confirmed aSAH for various reasons (such as no repeated DSA or CT, delayed presentation, no clipping/coiling, etc.). The incidence of AV in our study may be underestimated since we defined AV as moderate-to-sever arterial narrowing on DSA. A true incidence of AV, including all cases with mild, moderate and sever arterial narrowing on DSA, may be as high as 90% or even higher in both Caucasian and Asian patients [17, 27]. Additionally, we found 42.4% of patients who developed SV had cerebral infarction. The incidence proportion of cerebral infarction among SV cases ranged from 34.5%-80.0% in previous studies [5,28,29], depending on type and frequency of neuroimaging method used to detect cerebral infarction. We did not perform serial MRI or CT perfusion on scheduled basis, but rather CT scan on as-needed basis. MRI or CT perfusion is considered more sensitive to identify cerebral infarction compared to CT scan [30,31]. Performing serial MRI or CT/CT perfusion imaging may allow for early medical intervention and prevent progression to poor outcome in patients with aSAH, which should be recommended in clinical practice in China.

History of hypertension was associated with AV, SV, and cerebral infarction in our study. This is consistent with findings from another study conducted among Japanese aSAH patients [17]. However, studies conducted in Western developed countries reported inconsistent results on the role of hypertension on development of CVS [32–35]. Regardless of discrepancy between studies, hypertension is a modifiable risk factor of CVS in Chinese patients as suggested by our findings. Hypertension is usually not well controlled among patients in China; therefore, further study should be conducted to evaluate the impact of controlled hypertension on development of CVS after aSAH in Chinese patients. The modified Fisher grade, which accounts for thick cisternal and ventricular blood, is considered more accurate in predicting SV in aSAH patients [20, 36]. In our study, we found poor modified Fisher grade (3–4) was predictor of all three definitions of CVS, suggesting modified Fisher grade is more valuable tool to identify patients at risk of CVS than the original one, and it is applicable in Chinese patients.

Notably, we found patients from Uyghur ethnic group or other minorities were less likely to develop AV, SV or cerebral infarction, compared to those from Han ethic group. In previous studies, incidence of SV [37], cerebral infarction [32], or DCI [38] did not differ between whites and African Americans. However, a meta-analysis by Mocco J, et al. indicated Japanese patients with aSAH might be more likely to develop CVS than European patients [39], although the racial difference in incidence of CVS after aSAH in this meta-analysis could be due to some confounders such as smoking, hypertension, or variation in clinical condition of aSAH patients on admission, etc. In our study, the association of ethnicity with AV, SV, or cerebral infarction was still significant after adjustment for other clinical predictors of CVS, which may be due to genetic differences between ethnic groups in Xinjiang, China. However, it could be also due to confounders such as diabetes mellitus, alcohol intake, heart disease, cardiac function, etc. [4]. These potential confounding variables were not available in our study. Further research is needed to clarify the association of ethnicity and development of CVS in patients with aSAH in Western China.

Additionally, age ≥53 years, leukocyte count ≥11× 10^9^/L and current or former smokers before aSAH onset were independent risk factors of cerebral infarction in Chinese patients with aSAH. Similarly, most of previous studies have demonstrated a positive association of leukocytosis, age and history of smoking with the development of SV and cerebral infarction [30,40–43]. Furthermore, we found serum glucose level was associated with AV, but not with SV or cerebral infarction. Naidech AM and colleagues reported that moderate hypoglycemia was associated with SV, AV, cerebral infarction, and 3-month disability after subarachnoid hemorrhage [44]. This is only partially consistent with our finding. Additionally, we found gender, number of aneurysms, triglyceride level and aneurysm treatment were not associated with development of SV, or AV, or cerebral infarction. The lack of an association between these variables and SV, AV or cerebral infarction may be real in Chinese patients, or may be due to the retrospective nature of our study and other unknown confounders. Further research with sufficient sample size and prospective design is needed to confirm these associations.

### Limitations

This study has several limitations. Firstly, the design of our study is retrospective, and we only included patients with repeated neuroimaging findings, which may introduce selection bias. These patients without repeated neuroimaging findings are more likely to be aged ≥53 years (S1 Table). As such, we may underestimate the true incidence of cerebral infarction since elder age was associated with development of cerebral infarction in our study. Secondly, Although we restricted our definition of cerebral infarction on a CT scan to focus on lesions likely caused by CVS, we may include some ischemic lesions caused by other mechanisms (e.g. perforator vessel occlusion unnoticed at the time of surgical clipping or endovascular coiling, or delayed consequences of a dissection provoked during catheterization but undetected on an angiogram). Thirdly, some other factors which are potentially associated with SV, AV, or cerebral infarction (e.g. a history of diabetes mellitus, hydrocephalus, location of aneurysm, electrocardiogram changes, cardiac abnormalities, etc.) were not available in our study, which may confound the results of our study. Finally, aneurysm treatment (e.g. surgical clipping or endovascular coiling) was not done by the same surgeon. However, experience and skills of these surgeons who involved in aneurysm treatment were similar; therefore, we believe this may not introduce bias in our study.

### Conclusion

Our study demonstrated that a history of hypertension, poor modified Fisher grade (3–4), poor Hunt-Hess grade (4–5) and Han ethnicity were associated with increased risk of AV, SV, and cerebral infarction in Chinese patients with aSAH. Additionally, age ≥53 years, leukocyte count ≥11× 10^9^/L on admission and being current or former smokers were independent risk factors of cerebral infarction. Leukocyte count ≥11× 10^9^/L and aneurysm size ≥ 10 mm on admission were independent risk factors of SV. Serum glucose level ≥7.0 mmol/L on admission was independent risk factor of AV. We recommend early and aggressive therapy in these at-risk populations to prevent poor outcome in patients with aSAH. However, the associations observed in this study may not be applicable in general to Chinese patients with aSAH since nearly 37% of patients were excluded due to various reasons.

## Supporting Information

S1 Table Comparison of aSAH patients with complete data (n = 343) and those with missing data (n = 110) on repeated neuroimaging findings (DOCX)

## References

[1] Suarez JI, Tarr RW, Selman WR. Aneurysmal subarachnoid hemorrhage. N Engl J Med. 2006; 354(4):387–96. 10.1056/NEJMra052732 16436770

[2] Oyama K, Criddle L. Vasospasm after aneurysmal subarachnoid hemorrhage. 2004; 24(5):58–60. 15526491

[3] Pearl JD, Macdonald RL. Vasospasm after aneurysmal subarachnoid hemorrhage: need for further study. Acta Neurochir Suppl. 2008; 105: 207–10. 19066110 10.1007/978-3-211-09469-3_39

[4] Inagawa T. Risk Factors for Cerebral Vasospasm Following Aneurysmal Subarachnoid Hemorrhage: A Review of the Literature. World Neurosurg. 2016; 85:56–76. 10.1016/j.wneu.2015.08.052 26342775

[5] Kanamaru K, Suzuki H, Taki W. Cerebral Infarction After Aneurysmal Subarachnoid Hemorrhage. Acta Neurochir Suppl. 2016; 121:167–72. 10.1007/978-3-319-18497-5_30 26463943

[6] Roos YB, de Haan RJ, Beenen LF, Groen RJ, Albrecht KW, Vermeulen M. Complications and outcome in patients with aneurysmal subarachnoid haemorrhage: a prospective hospital based cohort study in the Netherlands. J Neurol Neurosurg Psychiatry. 2000; 68(3):337–41. 10.1136/jnnp.68.3.337 10675216 PMC1736841

[7] Frontera JA, Fernandez A, Schmidt JM, Claassen J, Wartenberg KE, Badjatia N, et al Defining vasospasm after subarachnoid hemorrhage: what is the most clinically relevant definition? Stroke. 2009; 40(6):1963–8. 10.1161/STROKEAHA.108.544700 19359629

[8] Kassell NF, Sasaki T, Colohan AR, Nazar G. Cerebral vasospasm following aneurysmal subarachnoid hemorrhage. Stroke. 1985; 16(4):562–72. 3895589 10.1161/01.str.16.4.562

[9] Etminan N, Vergouwen MD, Macdonald RL. Angiographic vasospasm versus cerebral infarction as outcome measures after aneurysmal subarachnoid hemorrhage. Acta Neurochir Suppl. 2013; 115:33–40. 10.1007/978-3-7091-1192-5_8 22890640

[10] Vergouwen MD, Vermeulen M, van Gijn J, Rinkel GJ, Wijdicks EF, Muizelaar JP, et al Definition of delayed cerebral ischemia after aneurysmal subarachnoid hemorrhage as an outcome event in clinical trials and observational studies: proposal of a multidisciplinary research group. Stroke. 2010; 41(10):2391–5. 10.1161/STROKEAHA.110.589275 20798370

[11] Vergouwen MD. Vasospasm versus delayed cerebral ischemia as an outcome event in clinical trials and observational studies. Neurocrit Care. 2011; 15(2):308–11. 10.1007/s12028-011-9586-8 21748502

[12] Zafar SF, Westover MB, Gaspard N, Gilmore EJ, Foreman BP, OʼConnor KL, et al Interrater Agreement for Consensus Definitions of Delayed Ischemic Events After Aneurysmal Subarachnoid Hemorrhage. J Clin Neurophysiol. 2016; 33(3):235–40. 10.1097/WNP.0000000000000276 27258447 PMC4894325

[13] Lysakowski C, Walder B, Costanza MC, Tramèr MR. Transcranial Doppler versus angiography in patients with vasospasm due to a ruptured cerebral aneurysm: A systematic review. Stroke. 2001; 32(10):2292–8. 11588316 10.1161/hs1001.097108

[14] Kumar G, Shahripour RB, Harrigan MR. Vasospasm on transcranial Doppler is predictive of delayed cerebral ischemia in aneurysmal subarachnoid hemorrhage: a systematic review and meta-analysis. J Neurosurg. 2016; 124(5):1257–64. 10.3171/2015.4.JNS15428 26495942

[15] Carrera E, Schmidt JM, Oddo M, Fernandez L, Claassen J, Seder D, et al Transcranial Doppler for predicting delayed cerebral ischemia after subarachnoid hemorrhage. Neurosurgery. 2009;65 (2):316–23 10.1227/01.NEU.0000349209.69973.88 19625911

[16] Kozak N, Bereczki D, Szabo S. Predictors of Symptomatic Vasospasm After Subarachnoid Hemorrhage: A Single Center Study of 457 Consecutive Cases. Turk Neurosurg. 2016; 26(4):545–9. 10.5137/1019-5149.JTN.14408-15.1 27400101

[17] Inagawa T, Yahara K, Ohbayashi N. Risk factors associated with cerebral vasospasm following aneurysmal subarachnoid hemorrhage. Neurol Med Chir (Tokyo). 2014; 54(6):465–73. 24670311 10.2176/nmc.oa.2013-0169 PMC4533446

[18] Harrod CG, Bendok BR, Batjer HH. Prediction of cerebral vasospasm in patients presenting with aneurysmal subarachnoid hemorrhage: a review. Neurosurgery. 2005; 56(4):633–54. 15792502 10.1227/01.neu.0000156644.45384.92

[19] Hunt WE, Hess RM. Surgical risk as related to time of intervention in the repair of intracranial aneurysms. J Neurosurg. 1968;28(1):14–20. 10.3171/jns.1968.28.1.0014 5635959

[20] Frontera JA, Claassen J, Schmidt JM, Wartenberg KE, Temes R, Connolly ES Jr, et al Prediction of symptomatic vasospasm after subarachnoid hemorrhage: the modified fisher scale. Neurosurgery. 2006; 59(1):21–7. 10.1227/01.NEU.0000218821.34014.1B 16823296

[21] Krejza J, Kochanowicz J, Mariak Z, Lewko J, Melhem ER. Middle cerebral artery spasm after subarachnoid hemorrhage: detection with transcranial color-coded duplex US. Radiology. 2005; 236(2):621–9. 10.1148/radiol.2362031662 16040918

[22] Charpentier C, Audibert G, Guillemin F, Civit T, Ducrocq X, Bracard S, et al Multivariate analysis of predictors of cerebral vasospasm occurrence after aneurysmal subarachnoid hemorrhage. Stroke. 1999; 30(7):1402–8. 10390314 10.1161/01.str.30.7.1402

[23] Kanamaru K, Suzuki H, Taki W. Risk factors for vasospasm-induced cerebral infarct when both clipping and coiling are equally available. Acta Neurochir Suppl. 2015; 120:291–5. 10.1007/978-3-319-04981-6_49 25366639

[24] Kumar A, Brown R, Dhar R, Sampson T, Derdeyn CP, Moran CJ, et al Early vs. delayed cerebral infarction after aneurysm repair after subarachnoid hemorrhage. Neurosurgery. 2013; 73(4):617–23. 10.1227/NEU.0000000000000057 23787882

[25] Wong GK, Leung JH, Yu JW, Lam SW, Chan EK, Poon WS, et al Early Cerebral Infarction after Aneurysmal Subarachnoid Hemorrhage. Acta Neurochir Suppl. 2016; 121:157–9. 10.1007/978-3-319-18497-5_28 26463941

[26] Jabbarli R, Reinhard M, Niesen WD, Roelz R, Shah M, Kaier K, et al Predictors and impact of early cerebral infarction after aneurysmal subarachnoid hemorrhage. Eur J Neurol. 2015; 22(6):941–7. 10.1111/ene.12686 25708292

[27] Crowley RW, Medel R, Dumont AS, Ilodigwe D, Kassell NF, Mayer SA, et al Angiographic vasospasm is strongly correlated with cerebral infarction after subarachnoid hemorrhage. Stroke. 2011; 42(4):919–23. 10.1161/STROKEAHA.110.597005 21350201

[28] Su FW, Lin YJ, Chang WN, Ho JT, Wang HC, Yang TM, et al Predictors and outcome of acute symptomatic cerebral infarctions following aneurysmal subarachnoid hemorrhage. J Neurol. 2010 2;257(2):264–70. 10.1007/s00415-009-5306-0 19756825

[29] Rabinstein AA, Weigand S, Atkinson JL, Wijdicks EF. Patterns of cerebral infarction in aneurysmal subarachnoid hemorrhage. Stroke. 2005; 36(5):992–7. 10.1161/01.STR.0000163090.59350.5a 15831836

[30] Korbakis G, Prabhakaran S, John S, Garg R, Conners JJ, Bleck TP, et al MRI Detection of Cerebral Infarction in Subarachnoid Hemorrhage. Neurocrit Care. 2016; 24(3):428–35. 10.1007/s12028-015-0212-z 26572141

[31] Turowski B, Schramm P. An Appeal to Standardize CT- and MR-Perfusion. Clin Neuroradiol. 2015; 25 Suppl 2:205–10. 26289412 10.1007/s00062-015-0444-5

[32] Macdonald RL, Rosengart A, Huo D, Karrison T. Factors associated with the development of vasospasm after planned surgical treatment of aneurysmal subarachnoid hemorrhage. J Neurosurg. 2003; 99(4):644–52. 10.3171/jns.2003.99.4.0644 14567598

[33] Fergusen S, Macdonald RL. Predictors of cerebral infarction in patients with aneurysmal subarachnoid hemorrhage. Neurosurgery. 2007; 60(4):658–67; 10.1227/01.NEU.0000255396.23280.31 17415202

[34] Kale SP, Edgell RC, Alshekhlee A, Borhani Haghighi A, Sweeny J, Felton J, et al Age-associated vasospasm in aneurysmal subarachnoid hemorrhage. J Stroke Cerebrovasc Dis. 2013; 22(1):22–7. 10.1016/j.jstrokecerebrovasdis.2011.05.024 21719308

[35] Yousef K, Crago E, Kuo CW, Horowitz M, Hravnak M, et al Predictors of delayed cerebral ischemia after aneurysmal subarachnoid hemorrhage: a cardiac focus. Neurocrit Care. 2010; 13(3):366–72. 10.1007/s12028-010-9408-4 20645025 PMC3131087

[36] Claassen J, Bernardini GL, Kreiter K, Bates J, Du YE, Copeland D, et al Effect of cisternal and ventricular blood on risk of delayed cerebral ischemia after subarachnoid hemorrhage: the Fisher scale revisited. Stroke. 2001; 32(9):2012–20. 11546890 10.1161/hs0901.095677

[37] Qureshi AI, Sung GY, Razumovsky AY, Lane K, Straw RN, Ulatowski JA. Early identification of patients at risk for symptomatic vasospasm after aneurysmal subarachnoid hemorrhage. Crit Care Med. 2000;28(4):984–90. 10809270 10.1097/00003246-200004000-00012

[38] Ko SB, Choi HA, Carpenter AM, Helbok R, Schmidt JM, Badjatia N, et al Quantitative analysis of hemorrhage volume for predicting delayed cerebral ischemia after subarachnoid hemorrhage. Stroke. 2011;42(3):669–74. 10.1161/STROKEAHA.110.600775 21257823

[39] Mocco J, Ransom ER, Komotar RJ, Mack WJ, Sergot PB, Albert SM, et al Racial differences in cerebral vasospasm: a systematic review of the literature. Neurosurgery. 2006; 58(2):305–14. 10.1227/01.NEU.0000195009.02412.E8 16462484

[40] McGirt MJ, Mavropoulos JC, McGirt LY, Alexander MJ, Friedman AH, Laskowitz DT, et al Leukocytosis as an independent risk factor for cerebral vasospasm following aneurysmal subarachnoid hemorrhage. J Neurosurg. 2003; 98(6):1222–6. 10.3171/jns.2003.98.6.1222 12816268

[41] Kasius KM, Frijns CJ, Algra A, Rinkel GJ. Association of platelet and leukocyte counts with delayed cerebral ischemia in aneurysmal subarachnoid hemorrhage. Cerebrovasc Dis. 2010; 29(6):576–83. 10.1159/000306645 20375501

[42] Fassbender K, Hodapp B, Rossol S, Bertsch T, Schmeck J, Schütt S, et al Inflammatory cytokines in subarachnoid haemorrhage: association with abnormal blood flow velocities in basal cerebral arteries. J Neurol Neurosurg Psychiatry. 2001; 70(4):534–7. 10.1136/jnnp.70.4.534 11254783 PMC1737308

[43] Lasner TM, Weil RJ, Riina HA, King JT Jr, Zager EL, Raps EC, et al Cigarette smoking-induced increase in the risk of symptomatic vasospasm after aneurysmal subarachnoid hemorrhage. J Neurosurg. 1997; 87(3):381–4. 10.3171/jns.1997.87.3.0381 9285602

[44] Naidech AM, Levasseur K, Liebling S, Garg RK, Shapiro M, Ault ML, et al Moderate Hypoglycemia is associated with vasospasm, cerebral infarction, and 3-month disability after subarachnoid hemorrhage. Neurocrit Care. 2010; 12(2):181–7. 10.1007/s12028-009-9311-z 19967566

