# Correction: Incidence and Predictors of Angiographic Vasospasm, Symptomatic Vasospasm and Cerebral Infarction in Chinese Patients with Aneurysmal Subarachnoid Hemorrhage

**DOI:** 10.1371/journal.pone.0172081

**Published:** 2017-02-08

**Authors:** The PLOS ONE Staff

The last two authors, Dangmurenjiafu Geng and Aisha Maimaitili, should be noted as contributing equally to this work. Additionally, the last author, Aisha Maimaitili, should also be indicated as a Corresponding Author. Dr. Maimaitili’s email is: mmtaili@aliyun.com.cn. The publisher apologizes for the error.
