# Supplementary material for: Incidence and Predictors of Angiographic Vasospasm, Symptomatic Vasospasm and Cerebral Infarction in Chinese Patients with Aneurysmal Subarachnoid Hemorrhage
Source: PLoS One. 2016 Dec 15;11(12):e0168657. doi: 10.1371/journal.pone.0168657 (PMC5158085; doi:10.1371/journal.pone.0168657)
Supplement: S1 Table — (DOCX) [file pone.0168657.s001.docx]

**S1 Table. Comparison of aSAH patients with complete data (n=343) and those with missing data (n=110) on repeated neuroimaging findings**

| **Category** | **Patient with complete data** | **Patient with missing data** | **χ^2^** | ***P*** |
| --- | --- | --- | --- | --- |
| **Gender** |  |  |  |  |
| **male** | 133(38.8) | 34(30.9) | 2.21 | 0.137 |
| **female** | 210(61.2) | 76(69.1) |  |  |
| **Age group** |  |  |  |  |
| **≥53 years** | 151(44.0) | 74(67.3) | 4.38 | 0.036 |
| **< 53 years** | 192(56.0) | 36(33.7) |  |  |
| **Ethnicity** |  |  |  |  |
| **Han** | 191(55.7) | 53(48.2) | 2.604 | 0.272 |
| **Uyghur** | 92(26.8) | 38(34.6) |  |  |
| **Others** | 60(17.5) | 19(28.2) |  |  |
| **History of cigarette smoking** |  |  |  |  |
| **Current or former smokers** | 64(18.7) | 21(19.1) | 0.010 | 0.920 |
| **Non-smokers** | 279(81.3) | 89(80.9) |  |  |
| **History of hypertension** |  |  |  |  |
| **Yes** | 149(43.7) | 47(42.7) | 0.017 | 0.896 |
| **No** | 194(56.3) | 63(57.3) |  |  |
| **Modified Fisher grade on admission** | |  |  |  |
| **0-2** | 194(56.6) | 59(53.6) | 0.289 | 0.591 |
| **3-4** | 149(43.4) | 51(46.4) |  |  |
| **Hunt-Hess grade on admission** | |  |  |  |
| **1-3** | 226(65.9) | 65(59.1) | 1.68 | 0.196 |
| **4-5** | 117(34.1) | 45(40.9) |  |  |
